# Molecular Landscape and Clinical Implication of *CCNE1-*amplified Esophagogastric Cancer

**DOI:** 10.1158/2767-9764.CRC-23-0496

**Published:** 2024-06-03

**Authors:** Naryan Rustgi, Sharon Wu, Timothy Samec, Phillip Walker, Joanne Xiu, Emil Lou, Sanjay Goel, Anwaar Saeed, Ryan H. Moy

**Affiliations:** 1 Department of Surgery, Division of Surgical Sciences, Columbia University Irving Medical Center, New York, New York.; 2 Caris Life Sciences, Phoenix, Arizona.; 3 Department of Medicine, Division of Hematology, Oncology, and Transplantation, University of Minnesota, Minneapolis, Minnesota.; 4 Department of Medicine, Division of Medical Oncology, Rutgers Cancer Institute of New Jersey, New Brunswick, New Jersey.; 5 Department of Medicine, Division of Hematology and Oncology, University of Pittsburgh Medical Center, Pittsburgh, Pennsylvania.; 6 Department of Medicine, Division of Hematology/Oncology, Columbia University Irving Medical Center, New York, New York.

## Abstract

Cyclin E overexpression as a result of *CCNE1* amplification is a critical driver of genomic instability in gastric cancer, but its clinical implication is largely unknown. Thus, we integrated genomic, transcriptomic, and immune profiling analysis of 7,083 esophagogastric tumors and investigated the impact of *CCNE1* amplification on molecular features and treatment outcomes. We identified *CCNE1* amplification in 6.2% of esophageal adenocarcinoma samples, 7.0% of esophagogastric junction carcinoma, 4.2% of gastric adenocarcinoma samples, and 0.8% of esophageal squamous cell carcinoma. Metastatic sites such as lymph node and liver showed an increased frequency of *CCNE1* amplification relative to primary tumors. Consistent with a chromosomal instability phenotype, *CCNE1* amplification was associated with decreased *CDH1* mutation and increased *TP53* mutation and *ERBB2* amplification. We observed no differences in immune biomarkers such as PD-L1 expression and tumor mutational burden comparing *CCNE1*-amplified and nonamplified tumors, although *CCNE1* amplification was associated with changes in immune populations such as decreased B cells and increased M1 macrophages from transcriptional analysis. Real-world survival analysis demonstrated that patients with *CCNE1*-amplified gastric cancer had worse survival after trastuzumab for HER2-positive tumors, but better survival after immunotherapy. These data suggest that *CCNE1*-amplified gastric cancer has a distinct molecular and immune profile with important therapeutic implications, and therefore further investigation of *CCNE1* amplification as a predictive biomarker is warranted.

**Significance:**

Advanced gastric cancer has a relatively dismal outcome with a 5-year overall survival of less than 10%. Furthermore, while comprehensive molecular analyses have established molecular subtypes within gastric cancers, biomarkers of clinical relevance in this cancer type are lacking. Overall, this study demonstrates that *CCNE1* amplification is associated with a distinct molecular profile in gastric cancer and may impact response to therapy, including targeted therapy and/or immunotherapy.

## Introduction

Gastric adenocarcinoma is one of the most common cancers worldwide, with over 1 million new diagnoses and 700,000 deaths annually (1, 2). Comprehensive molecular analyses have identified key genetic alterations in gastric cancer and defined four molecular subtypes: genomically stable (GS), chromosomal instability (CIN), microsatellite instability (MSI), and Epstein-Barr virus positive (RRID:SCR_003193). While genomic profiling of patient tumors has become commonplace, clinically actionable biomarkers for gastric cancer treatment are limited to HER2 positivity/amplification, microsatellite instability-high (MSI-H) status, and PD-L1 overexpression. Immunotherapy with PD1 inhibitors such as nivolumab and pembrolizumab are now approved in combination with first-line chemotherapy, but the benefit is modest and median survival remains between 1 and 2 years (3–6). Recent data suggest that the Claudin 18.2 antibody zolbetuximab improves outcomes when added to chemotherapy in patients with Claudin 18.2-positive tumors (∼30%–40% of gastric cancers); however, this agent is not yet approved and improves median overall survival (OS) by less than 3 months (7–9). Therefore, there is a pressing need to define additional clinically relevant biomarkers in patients with gastric cancer.

One alteration that may impact therapeutic response is overexpression of the cell cycle regulator cyclin E, typically through copy-number amplification of the gene *CCNE1*. Amplification of *CCNE1* has been identified in diverse tumor types including approximately 12% of esophagogastric cancer (EGC) and 20% of high-grade serous ovarian cancers from The Cancer Genome Atlas (TCGA) analysis (10). *CCNE1* amplification promotes unscheduled S-phase entry, DNA replication stress, and CIN (11–14). Previous studies identified a correlation between *CCNE1* amplification and increased liver metastases as well as poor survival in select cohorts of patients with gastric cancer (15, 16). *CCNE1* has been found to be commonly coaltered with *ERBB2* amplification/HER2 overexpression, and preclinical and clinical studies suggest that *CCNE1* amplification promotes resistance to HER2-targeted therapies (i.e., trastuzumab and lapatinib; refs. 17, 18). Moreover, immunologically “cold” esophagogastric (EG) adenocarcinoma with low T-cell abundance demonstrated enrichment of *CCNE1* amplification, suggesting that *CCNE1* amplification may promote immune resistance (19). However, the clinical impact of *CCNE1* amplification in a real-world population is unknown. Here, we performed detailed molecular profiling of *CCNE1*-amplified EGC to understand the genomic and immune landscape of these tumors and define treatment outcomes.

## Materials and Methods

### Tissue Acquisition

Tumor tissue from patients diagnosed with esophageal squamous cell carcinoma (ESCC), esophageal adenocarcinoma (EAC), esophagogastric junction carcinoma (EJC), or gastric adenocarcinoma were obtained from surgical or biopsy specimens. Tumors underwent comprehensive molecular analysis at Caris Life Sciences. This study was conducted in accordance with guidelines of the Declaration of Helsinki, Belmont report, and U.S. Common rule.

### Next-generation Sequencing

Next-generation sequencing (NGS) was performed on genomic DNA isolated from formalin-fixed paraffin-embedded (FFPE) tumor samples using NextSeq or NovaSeq (Illumina, Inc.) at Caris Life Sciences. A custom-designed SureSelect XT assay was used to enrich 592 whole-gene targets (Agilent Technologies). Variants were detected with >99% confidence based on allele frequency and amplicon coverage, with an average sequencing depth of coverage of >500× and an analytic sensitivity of 5%. For whole-exome sequencing (WES) using NovaSeq, a hybrid pull-down panel of baits designed to enrich for more than 700 clinically relevant genes at high coverage (>500×) and high read-depth was used, along with another panel designed to enrich for an additional >20,000 genes at lower depth (>250×). Prior to molecular profiling, tumor enrichment was attained by manual microdissection techniques. Genetic variants identified were interpreted by board-certified molecular geneticists and categorized according to the American College of Medical Genetics and Genomics standards. When assessing mutation frequencies of individual genes, “pathogenic,” and “likely pathogenic” were counted as mutations while “benign,” “likely benign” variants and “variants of unknown significance” were excluded. Tumor mutational burden (TMB) was measured by totaling somatic mutations per tumor (high >10 mt/Mb). A copy number (CN) cutoff of CN ≥ 6 was used to define gene amplification. CN gain was defined as CN ≥ 3 and CN < 6. The CN cutoff of 6 for amplification was determined internally at Caris as a standard (based on *MYC/ERBB2* and validated with IHC).

### Whole Transcriptome Sequencing

FFPE specimens underwent pathology review to diagnose percent tumor content and tumor size; a minimum of 10% of tumor content in the area for microdissection was required to enable enrichment and extraction of tumor-specific RNA. Qiagen RNA FFPE tissue extraction kit was used for extraction to detect fusions and the RNA quality and quantity were determined using the Agilent TapeStation. Biotinylated RNA baits were hybridized to the synthesized and purified cDNA targets and the bait–target complexes were amplified by PCR. The libraries were quantified, normalized and the pooled libraries were denatured, diluted, and sequenced; the reference genome used was GRCh37/hg19. Transcripts per million molecules were generated using the Salmon expression pipeline for transcription counting. Immune cell fraction was calculated by quanTIseq (20).

### IHC and Chromogenic *In Situ* Hybridization

IHC of PD-L1 via 22C3 antibody; MLH1, M1 antibody; MSH2, G2191129 antibody; MSH6, 44 antibody; PMS2, EPR3947 antibody; and HER2 via 4B5 antibody (Ventana Medical Systems, Inc.) were performed on full FFPE sections of glass slides. Slides were stained using automated staining techniques per the manufacturer's instructions (Ventana), and were optimized and validated per Clinical Laboratory Improvement Amendments/The College of American Pathologists (CAP) and International Organization for Standardization requirements. Staining was scored for intensity (0 = no staining; 1+ = weak staining; 2+ = moderate staining; 3+ = strong staining) and staining percentage (0%–100%). The complete absence of protein expression of any of the four proteins tested (0+ in 100% of cells) was considered deficient mismatch repair proficiency (MMR). Combined positive score (CPS) ≥ 1 was deemed positive for PD-L1 analysis. A subset of tumors was tested for HER2 by chromogenic *in situ* hybridization (CISH; INFORM DUAL HER2 ISH Assay, Ventana), and HER2 status was interpreted following American Society of Clinical Oncology/CAP scoring criteria (21). A board-certified pathologist evaluated all IHC and CISH results independently.

### MSI/MMR Status

A combination of multiple test platforms was used to determine the MSI or MMR status of the tumors profiled, including fragment analysis (FA, Promega), IHC, and NGS (7,000 target microsatellite loci were examined and compared with the reference genome hg19 from the University of California, Los Angeles, CA). The three platforms generated highly concordant results, as reported previously. In the rare cases of discordant results, the MSI or MMR status of the tumor was determined in the order of IHC, FA, and NGS (22).

### TCGA Database Access

Data from TCGA were accessed and utilized as a comparative tool for currently known and analyzed gene modifications in gastric adenocarcinoma. Accession was completed using TCGA webpage (RRID:SCR_003193).

### CODEai

Real-world OS (rwOS) information was obtained from insurance claims data and calculated from first of treatment time to last patient contact. Kaplan–Meier estimates were calculated for molecularly defined patient cohorts across the time period determined by sample collection or first treatment through last patient contact.

### Statistical Analysis

Statistical significance was determined using the *χ*^2^, Fisher exact, or Mann–Whitney test, as appropriate. The Benjamini–Hochberg method was implemented to adjust *P* values for multiple comparisons and a *q* ≤ 0.05 was regarded as statistically significant to reduce false discovery rate. rwOS was compared between groups using the log-rank test.

### Ethics Approval

This study was conducted in accordance with the guidelines of the Declaration of Helsinki, Belmont report, and U.S. Common rule. In keeping with 45 CFR 46.101(b) (4), this study was performed utilizing retrospective, deidentified clinical data. Therefore, this study was considered Institutional Review Board exempt, and no patient consent was necessary from the subject.

### Data Availability

The datasets generated and/or analyzed during the current study are available from the corresponding author on reasonable request. The NGS raw data are owned by Caris Life Sciences and cannot be publicly shared because of the data usage agreement signed by Dr. Ryan H. Moy. Qualified researchers can apply for access to these data by contacting Joanne Xiu (jxiu@carisls.com) and signing a data usage agreement.

## Results

### Patient Demographics and CCNE1 Amplification Status

**FIGURE 1:**
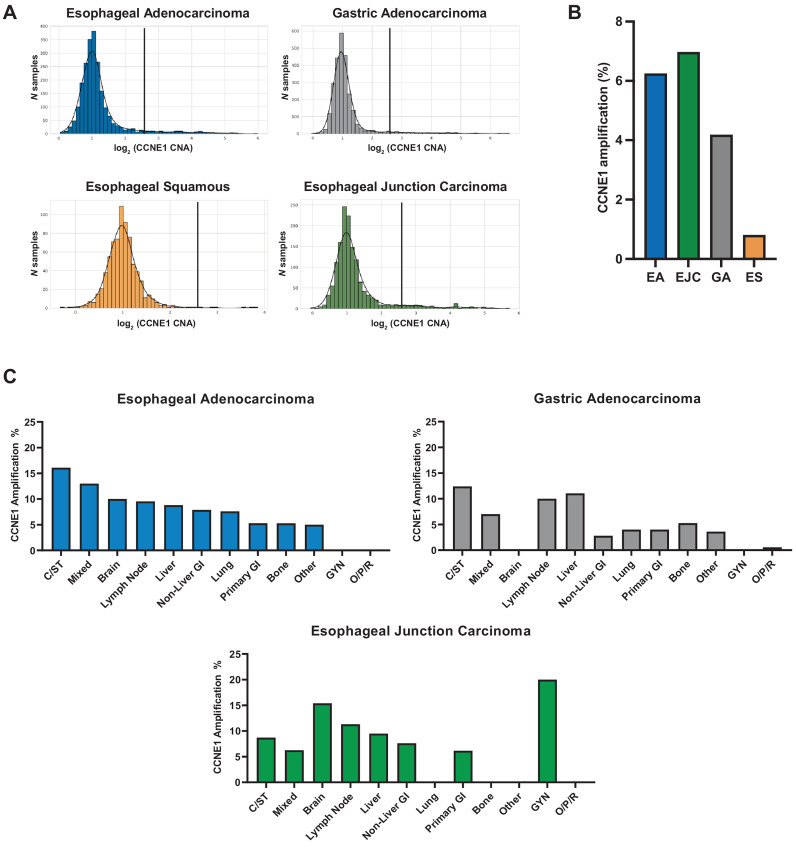
Incidence of *CCNE1* amplification in EGC by real-world analysis. NGS, including targeted and WES, was performed on 7,083 patients including 751 patients with ESCC, 2,276 patients with EA, 1,449 patients with EJC, and 2,607 patients with gastric adenocarcinoma (**A**). CN distribution of *CCNE1* is reported by total numbers for EAC, ESCC, gastric adenocarcinoma, and EJC. *CCNE1* amplification is defined as a CN ≥ 6 which is denoted by the black line. Percent *CCNE1* amplification across cancer types (**B**). *CCNE1* amplification by site, either primary tumor or metastasis, is shown for EAC, gastric adenocarcinoma, and EJC (**C**).

The study population was composed of 7,083 patients including 751 patients with ESCC, 2,276 patients with esophageal adenocarcinoma (EA), 1,449 patients with EJC, and 2,607 patients with gastric adenocarcinoma. Baseline patient characteristics are summarized in Table 1. We performed NGS including targeted and WES to identify the mutations and CN alterations (CNA) in each tumor sample, using a cutoff of *CCNE1* CN≥6 to define *CCNE1* amplification. Compared with adenocarcinoma, ESCC demonstrated a narrower range of *CCNE1* amplification (Fig. 1A). We identified *CCNE1* amplification in 6.2% of EA, 7.0% of EJC, and 4.2% of gastric adenocarcinoma samples; by contrast, *CCNE1* was rarely amplified in ESCC (0.8%; Fig. 1B). The median *CCNE1* CN was 2.05, 2.05, 1.97, and 1.98 for EA, EJC, gastric adenocarcinoma, and ESCC, respectively. We compared the *CCNE1* amplification rate in untreated tumors versus tumors exposed to prior therapy and observed a higher frequency of *CCNE1* amplification in untreated EA but no difference in the other EGC subtypes (Supplementary Fig. S1), suggesting that *CCNE1* amplification is likely present at diagnosis prior to therapy.

**TABLE 1:** Patient demographic data and frequency of *CCNE1* amplification by EGC subtype

	Esophageal adenocarcinoma		Esophageal squamous cell carcinoma		Esophagogastric junction carcinoma		Gastric adenocarcinoma	
Characteristic	Amp	No Amp	Amp	No Amp	Amp	No Amp	Amp	No Amp
*N*	142 (6.24)	2,134 (93.8)	6 (0.80)	745 (99.2)	101 (6.97)	1,348 (93)	109 (4.18)	2,498 (95.8)
Age, median (range)	62.5 (23–90)	66 (14–90)	63.5 (59–87)	67 (30–90)	59 (31–84)	66 (19–90)	66 (28–90)	65 (15–90)
Gender								
Female	15 (10.6)	282 (13.2)	3 (50)	254 (34.1)	15 (14.9)	254 (18.8)	36 (33)	1,009 (40.4)
Male	127 (89.4)	1,852 (86.8)	3 (50)	491 (65.9)	86 (85.1)	1,094 (81.2)	73 (67)	1,489 (59.6)
Site, *N* (%)								
Primary	79 (55.6)	1418 (66.4)	2 (33.3)	557 (74.8)	40 (39.6)	610 (45.3)	68 (62.4)	1,664 (66.6)
Metastatic	59 (41.5)	687 (32.2)	4 (66.7)	181 (24.3)	59 (58.4)	699 (51.9)	39 (35.8)	791 (31.7)
Unclear	4 (2.8)	29 (1.4)	0 (0)	7 (0.9)	2 (2)	39 (2.9)	2 (1.8)	43 (1.7)

There were 4,438 samples from primary tumors (63%) and 2,591 samples from metastases (37%). Compared with primary tumors, metastatic sites tended to have a higher frequency of *CCNE1* amplification (Fig. 1C; Supplementary Table S1). The frequency of *CCNE1* amplification in EA primary tumors was 5.3% versus 9.1% in metastatic samples (*P* = 0.0061, Fisher exact test), with an amplification rate of 7.6% in lung metastases, 8.8% in liver metastases, 9.5% in lymph node metastases, 10% in brain metastases, and 16.1% in connective/soft tissue metastases. Similarly, we observed a higher frequency of *CCNE1* amplification in gastric adenocarcinoma metastatic sites (6.0%) such as liver and lymph node metastases (11.1% and 10.4%, respectively) compared with primary tumors (3.9%; *P* = 0.037, Fisher exact test). These data suggest that *CCNE1* amplification is common in EG adenocarcinomas, particularly in metastatic lesions.

### Genomic Coalterations and Oncologic Biomarker Prevalence in Concordance with CCNE1^Amp^

**FIGURE 2:**
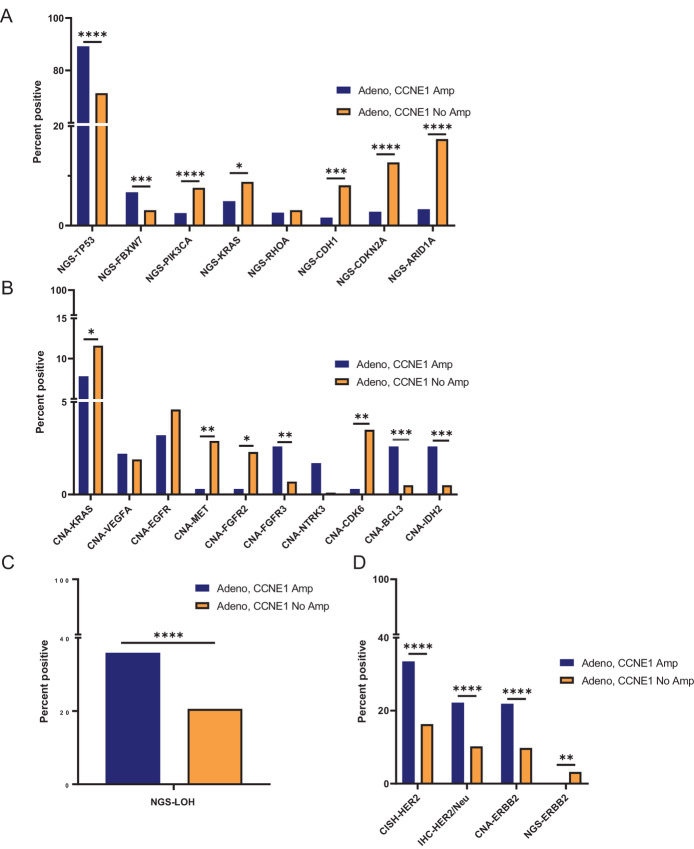
Frequently occurring molecular coalterations in *CCNE1*-amplified EGC. Frequency of comutated genes among all *CCNE1-*amplified and nonamplified EG adenocarcinoma by WES and NGS (**A**). CNAs co-occurring with *CCNE1* amplification (**B**). LOH with *CCNE1* amplification (**C**). HER2 overexpression or *ERBB2* amplification in CCNE1-amplified EG adenocarcinoma by CISH assays, IHC, or NGS (**D**). Statistical significance is displayed as the following: *, *q* < 0.05; **, *q* < 0.01; ***, *q* < 0.001; ****, *q* < 0.0001.

Using WES data and IHC, we next assessed for common coalterations with *CCNE1* amplifications and compared the molecular profiles of *CCNE1*-amplified and nonamplified EG adenocarcinoma. We observed several differences in critical oncogenes and tumor suppressors. The most common comutated gene across EG adenocarcinoma was *TP53,* consistent with *CCNE1*-amplified EG adenocarcinoma being most associated with the CIN molecular subtype (Fig. 2A; Supplementary Fig. S2A–S2C). Here we observed that *TP53* mutations were significantly enriched in *CCNE1*-amplified gastric adenocarcinoma (87.0% vs. 54.6%, *P* = 3.2 × 10^11^) but only slightly increased in *CCNE1*-amplified EA (90.6% vs. 85.6%, *P* = 0.10; Supplementary Fig. S2A and S2C). We also observed increases in *FBXW7* mutations in *CCNE1*-amplified EG adenocarcinoma, with a significant increase in *CCNE1*-amplified EA (8.9% vs. 2.4%, *P* = 0.002) and modest albeit not statistically significant increase in *CCNE1*-amplified gastric adenocarcinoma (5% vs. 3.4%, *P* = 0.39; Fig. 2A; Supplementary Fig. S2). Interestingly, FBXW7 encodes an ubiquitin ligase complex that is demonstrated to negatively posttranslationally regulate a multitude of critical proteins, including CCNE1 (23).

From TCGA analysis, several frequently occurring alterations have been described in GS, gastric adenocarcinoma, including mutations in *CDH1* and *RHOA*, as well as *CLDN18:ARHGAP* fusions. We observed a significant reduction in *CDH1* mutations in *CCNE1*-amplified gastric adenocarcinoma versus nonamplified gastric adenocarcinoma (2.9% vs. 16.4%, *P* = 0.0002) as well as a reduction in *CLDN18:ARHGAP* fusions (0% vs. 6.8%); however, the frequency of *RHOA* mutations were similar (Supplementary Fig. S2C). We also observed frequent *PIK3CA* mutations, associated with the EBV+ subtype, in gastric adenocarcinoma and EA, with a decrease in *CCNE1*-amplified versus nonamplified gastric adenocarcinoma (1.9% vs. 9.2%, *P* = 0.01) and a similar frequency in *CCNE1*-amplified versus nonamplified EA. Finally, we observed a trend toward significant decreases in *ARID1A* and *CDKN2A* mutations across *CCNE1*-amplified EG adenocarcinoma.

CNA analysis revealed CNA differences in several genes in *CCNE1*-amplified EG adenocarcinoma (Fig. 2B). We observed a significant increase in CNA in *FGFR3*, *BCL3*, and *IDH2* in *CCNE1*-amplified versus nonamplified EG adenocarcinoma (Fig. 2B). Other CNA (besides *ERBB2*) which have been investigated as potential driver alterations or targetable biomarkers demonstrated reduced frequency in *CCNE1*-amplified EG adenocarcinoma including *KRAS* (7.8% vs. 11.6%, *P* = 0.32), *EGFR* (3.2% vs. 4.6%, *P* = 0.22), *MET* (0.3% vs. 2.9%, *P* = 0.004), and *FGFR2* (0.3% vs. 2.3%, *P* = 0.013), suggesting that *CCNE1* amplification is molecularly distinct from these other drivers. LOH is a frequent genetic event in many cancers and is a hallmark of CIN. We leveraged WES data to measure genomic LOH and observed a significant increase in LOH in *CCNE1*-amplified EG adenocarcinoma (35.9% vs. 20.6%, *P* = 3.1 × 10^6^; Fig. 2C).

Previous studies have identified concomitant focal amplification of *CCNE1* and *ERBB2* in gastric cancer as well as breast cancer (24, 25). Consistent with these data, we also observed a significant increase in HER2 overexpression or *ERBB2* amplification in *CCNE1*-amplfiied EG adenocarcinoma by either CISH, IHC, or NGS (Fig. 2D). By contrast, *ERBB2* mutations were reduced in *CCNE1*-amplified EG adenocarcinoma.

To further determine whether CN level correlates with differential genomic features, we compared tumors with *CCNE1* CN≥6 (amplification), CN≥3 and <6 (gain), and CN<3 (neutral; Supplementary Fig. S3A–S3D). Interestingly, we observed stepwise differences in select alterations based on CN level. For example, EA with *CCNE1* gain demonstrated intermediate frequency of *TP53*, CDKN2A, *ARIDA*, and *ERBB2* alterations relative to *CCNE1* neutral and amplified tumors, while gastric adenocarcinoma with *CCNE1* gain showed intermediate levels of *TP53* mutation, *CDH1* mutation, *KMT2D* mutation, LOH, and *ERBB2* amplification. These data suggest there may be differences in molecular phenotype based on the magnitude of *CCNE1* CNA.

We compared our findings of *CCNE1* amplification rates in EGC with the Memorial Sloan Kettering (MSK) *Cancer Discovery* 2017 cohort (26) [publicly available in cBioPortal (27, 28)] (Supplementary Fig. S4A). Among 341 esophagogastric tumors that underwent MSK-IMPACT sequencing, 9.2% (32 samples) harbored *CCNE1* amplification, including 13/147 (8.8%) gastric adenocarcinoma samples, 12/137 (8.8%) EAC samples, and 7/57 (12.3%) EJC samples. While limited by the smaller sample size, examination of coalterations among the *CCNE1*-amplified versus non–*CCNE1*-amplified cohorts revealed trends toward increased *FBXW7* mutation (9.4% vs. 3.2%, *P* = 0.11) increased *ERBB2* amplification (43.8% vs. 27.5%, *P* = 0.066), decreased *CDH1* mutation (0% vs. 6.47%, *P* = 0.24), decreased *CDKN2A* mutation (3.1% vs. 12.0%, *P* = 0.23), and decreased *ARID1A* mutation (0% vs. 14.2%, *P* = 0.022; Supplementary Fig. S4B). Overall, these findings are largely consistent with comprehensive genomic profiling from our larger real-world cohort and indicate that *CCNE1*-amplified EG adenocarcinoma harbors a distinct molecular landscape compared with nonamplified tumors.

### Immune Microenvironment and Transcriptional Landscape of CCNE1^Amp^ EGC

**FIGURE 3:**
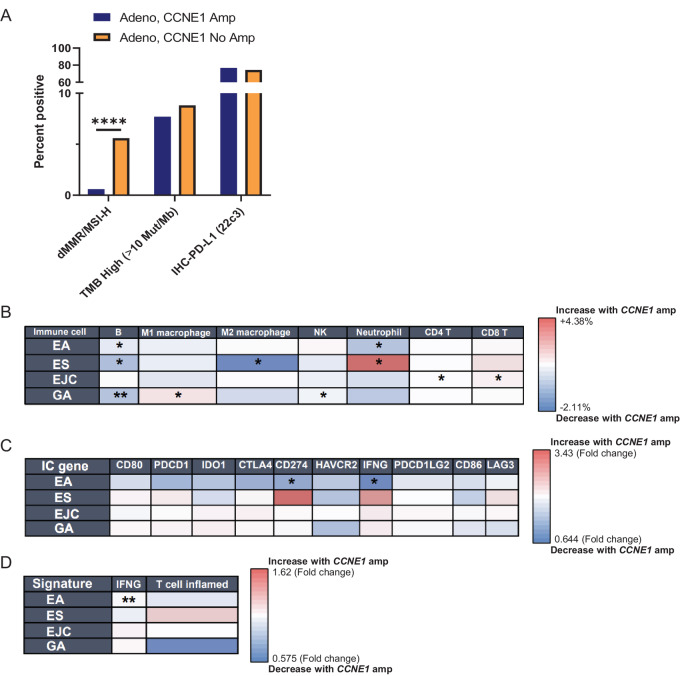
Immune microenvironment and biomarker analysis of *CCNE1*-amplified EGC. Breakdown of immune biomarkers of *CCNE1*-amplified versus nonamplified EG adenocarcinoma by MMR/MSI-H status, PD-L1 (22C3) expression, and TMB high status (**A**). Characterization of percentage infiltration of specified immune cell populations in *CCNE1*-amplified versus nonamplified EGC from WTS by RNA deconvolution analysis (**B**). Expression of immune-related genes in *CCNE1*-amplified versus nonamplified from RNA sequencing (**C**). Immune-related gene signatures including IFNG and T cell–inflamed signatures (**D**). Statistical significance is displayed as the following: *, *q* < 0.05; **, *q* < 0.01; ***, *q* < 0.001; ****, *q* < 0.0001.

CIN has been linked to immune cell exclusion, and prior analysis of tumors within TCGA demonstrated that “immune-cold” CIN-type EG adenocarcinoma are enriched for *CCNE1* amplifications, correlating with low CD8^+^ T-cell abundance (19). Therefore, we examined immune-related biomarkers in *CCNE1*-amplified EGC. We observed a significant decrease in MMR/MSI-H tumors in *CCNE1*-amplified adenocarcinoma (0.6% vs. 5.6%, *P* = 5.0 × 10^−5^), while there were no significant differences in TMB-high status (≥10 mt/Mb) between *CCNE1*-amplified and nonamplified tumors (Fig. 3A). Median TMB for both *CCNE1*-amplified and nonamplified tumors was 4.0. PD-L1 is a validated biomarker, with higher expression by CPS tending to confer higher responsiveness to anti-PD1 inhibitors (29), but we found no difference in the percentage of PD-L1–positive (CPS ≥1) or mean PD-L1 CPS (Fig. 3A).

Next, we utilized RNA deconvolution of whole transcriptome sequencing (WTS) to characterize the immune microenvironment of *CCNE1*-amplified tumors relative to nonamplified EGC (Fig. 3B–D; Supplementary Fig. S5). In *CCNE1*-amplified EA, we observed a statistically significant underrepresentation of B cells and neutrophils (Fig. 3B). Similarly, we found that *CCNE1*-amplified gastric adenocarcinoma harbored decreased B cells in addition to natural killer cells, whereas M1 macrophages showed increased representation. There were no significant differences in CD4^+^ or CD8^+^ T-cell representation in *CCNE1*-amplified versus nonamplified EA or gastric adenocarcinoma. Assessment of individual immune-related genes demonstrated decreased *IFNG* and *CD274* RNA expression in *CCNE1-*amplified EA (Fig. 3C). Finally, we analyzed specific immune-related gene signatures and found decreased *IFN* signatures in *CCNE1*-amplified EA, as well as trends toward decreased T cell–inflamed signatures in *CCNE1*-amplified EA and gastric adenocarcinoma (Fig. 3D).

In addition to immune microenvironment deconvolution, we performed ingenuity pathway analysis of differentially expressed genes. Consistent with the role of CCNE1 overexpression in driving cell cycle progression, we observed upregulation of pathways related to the cell cycle including kinetochore metaphase signaling and G_1_–S checkpoint regulation (Supplementary Fig. S6). One of the most strongly overrepresented pathways in both *CCNE1-*amplified gastric adenocarcinoma and EA was liver X receptor/retinoid X receptor activation, which has been linked to regulation of tumor growth, metastasis, and restriction of innate immune suppression in tumors (30–32). Together, these data indicate that *CCNE1*-amplified EG adenocarcinoma harbors a distinct immune and transcriptional landscape.

### Treatment Outcome and CODEai Survival Analysis of CCNE1^Amp^ EGC

**FIGURE 4:**
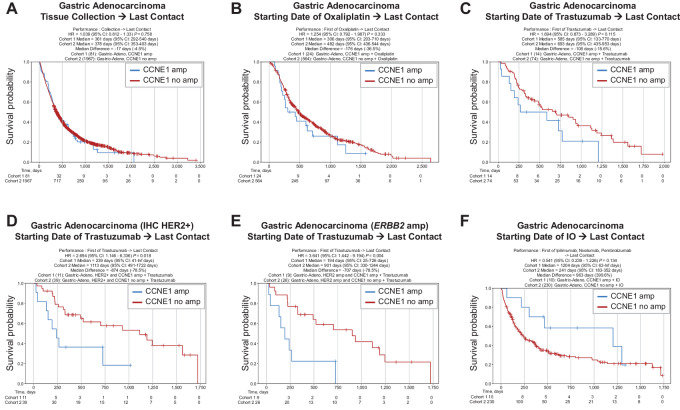
rwOS in patients with *CCNE1*-amplified gastric adenocarcinoma. OS (calculated from tissue collection to last day of contact) of *CCNE1*-amplified versus nonamplified gastric adenocarcinoma (**A**). Comparison of survival of *CCNE1*-amplified versus nonamplified gastric adenocarcinoma treated with oxaliplatin (calculated from start of treatment to last day of contact; **B**). Comparison of survival of *CCNE1*-amplified versus nonamplified gastric adenocarcinoma treated with trastuzumab (**C**). Comparison of survival of *CCNE1*-amplified versus nonamplified HER2-positive gastric adenocarcinoma (by IHC) treated with trastuzumab (**D**). Comparison of survival of *CCNE1*-amplified versus nonamplified HER2-positive gastric adenocarcinoma (by *ERBB2* CNA analysis) treated with trastuzumab (**E**). Comparison of survival of *CCNE1*-amplified versus nonamplified gastric adenocarcinoma treated with immunotherapy with a PD1 or PD-L1 inhibitor (**F**).

We utilized our real world evidence (RWE) database to analyze treatment history and survival outcomes, comparing patients with *CCNE1*-amplified versus nonamplified tumors. Patients with *CCNE1*-amplified EA had prolonged OS (defined as the time from tissue collection to last day of contact) compared with patients with non–*CCNE1*-amplified EA [HR = 0.756, 95% confidence interval (CI): 0.605–0.945, *P* = 0.014] (Supplementary Fig. S7A); similar results were observed in patients with EJC (Supplementary Fig. S7D). While there were no differences in OS in patients with *CCNE1*-amplified gastric adenocarcinoma (Fig. 4A), interestingly, we observed differences in survival outcomes after receiving specific therapies. There were no differences in survival after treatment with oxaliplatin (Fig. 4B); however, compared with patients with non–*CCNE1*-amplified gastric adenocarcinoma, patients with *CCNE1*-amplified gastric adenocarcinoma showed a trend toward shorter survival after receiving trastuzumab (HR = 1.694, 95% CI: 0.873–3.289, *P* = 0.115; Fig. 4C). When limiting analysis to only patients with HER2 positivity by IHC (HR = 2.694, 95% CI: 1.146–6.336, *P* = 0.018) or NGS (HR = 3.641, 95% CI: 1.442–9.194, *P* = 0.004), patients with HER2+/*CCNE1*-amplified gastric adenocarcinoma demonstrated significantly worse survival after trastuzumab compared with patients with HER2+/non–*CCNE1*-amplified gastric adenocarcinoma (Fig. 4D and E). In contrast, patients with *CCNE1*-amplified gastric adenocarcinoma exhibited a trend toward improved survival after receiving immunotherapy with a PD1 or PD-L1 inhibitor (HR = 0.541, 95% CI: 0.239–1.226, *P* = 0.134; Fig. 4F). These differences in survival outcomes with trastuzumab or immunotherapy were limited to *CCNE1*-amplified gastric adenocarcinoma, as we did not find any significant survival differences in *CCNE1*-amplified EA or EJC after trastuzumab or immunotherapy, although there was a slight trend toward improved survival after immunotherapy in *CCNE1*-amplified EJC (Supplementary Fig. S7B, S7C, S7E, and S7F). Although these analyses are limited by the small sample size of patients with available survival data, these data suggest that *CCNE1* amplification may be associated with clinical outcomes in response to either HER2-targeted therapy or immunotherapy, specifically in patients with gastric adenocarcinoma.

## Discussion

*CCNE1* amplification is a common alteration in multiple tumor types that leads to G_1_–S-phase checkpoint dysregulation and genomic instability, a key hallmark of cancer. While *CCNE1* amplification has been previously identified in gastric cancer, its clinical relevance in a real-world setting has been largely uncharacterized. In this study, we utilized comprehensive molecular profiling to define the incidence and molecular features of *CCNE1*-amplified gastric cancer, as well as implications on treatment outcome.

We identified *CCNE1* amplification in approximately 7% of EA and 4% of gastric adenocarcinoma. This frequency is lower than previous studies, in which *CCNE1* amplification has been reported in 10%–12% of EGCs (10, 15, 33). One difference may be the cutoff used to define gene amplification, as some studies have utilized a CN cutoff of 4 as opposed to our study in which we used a threshold of 6. In addition, the histologic makeup of the study population affects the overall frequency of *CCNE1* amplification, which is more likely to be associated with intestinal-type gastric cancer than diffuse type. Finally, the tissue site may impact the frequency of *CCNE1* amplification, as we observed a higher percentage of *CCNE1* amplification in metastatic sites compared with primary tumors. The association of *CCNE1* amplification with metastases is consistent with other data suggesting that patients with *CCNE1*-amplified gastric cancer are more likely to have liver metastases (15). Human metastases have also been shown to be enriched for CIN, and genetic inhibition of CIN delayed metastatic in preclinical models, suggesting that CIN promotes metastatic progression (34–36). Our data support the notion that *CCNE1* amplification is a common alteration in EG adenocarcinoma and may correlate with increased metastatic potential.

We assessed common coalterations with *CCNE1* amplification and observed frequent *TP53* mutations as well as decreased *CDH1* mutation. Moreover, *CCNE1*-amplified tumors tended to have a greater number of CNAs, including an enrichment for HER2 overexpression/*ERBB2* amplification as has been described previously. These data are consistent with *CCNE1* amplification as a critical driver of CIN and its association with CIN-type gastric cancer.

Importantly, we found that *CCNE1* amplification may have therapeutic relevance in gastric cancer. Previous studies have suggested that *CCNE1* amplification is a potential prognostic factor in certain tumor types. For example, *CCNE1* amplification has been associated with poor survival in patients with triple-negative breast cancer (37) and ovarian cancer (10, 38). In a small cohort of patients with resected gastric cancer, *CCNE1* overexpression was associated with worse disease-free survival (16). Furthermore, *CCNE1* expression has been suggested to be a predictive biomarker, with high *CCNE1* RNA expression correlating with decreased progression-free survival in patients with metastatic hormone receptor–positive, HER2-negative breast cancer receiving the CDK4/6 inhibitor Palbociclib (39). *CCNE1* amplification has also been linked to therapeutic resistance to trastuzumab in breast cancer and gastric cancer (18, 24). In addition, in a phase II trial of chemotherapy plus the HER2 inhibitor lapatinib for gastric cancer, nonresponders were enriched for *CCNE1* amplification (17). Using insurance claims data, we found that *CCNE1* amplification was not associated with OS in gastric cancer. However, patients with HER2-positive gastric cancer and concurrent *CCNE1* amplification experienced poorer prognosis after receiving trastuzumab than those without concurrent *CCNE1* amplification. This is suggestive of *CCNE1* amplification as a predictive biomarker and further demonstrates that *CCNE1* amplification may be one mechanism that decreases efficacy or promotes resistance to targeted therapies. Indeed, recent studies suggest that *CCNE1* amplification may predict sensitivity to the Wee1 kinase inhibitor adavosertib (40). Modulation of CCNE1 expression in HER2-positive cancer cell lines regulated sensitivity to the HER2-targeting antibody–drug conjugate trastuzumab deruxtecan (T-DXd) *in vitro*, and adavosertib acted synergistically with T-DXd in HER2-expressing patient-derived xenografts *in vivo* (41).

While we observed impaired survival after trastuzumab in *CCNE1*-amplified gastric cancer compared with nonamplified gastric adenocarcinoma, surprisingly, we noted a trend toward improved survival after initiation of immunotherapy. In addition to promoting genomic instability, *CCNE1* amplification may also modulate the tumor-immune microenvironment. On one hand, CIN can stimulate inflammatory pathways, such as through generation of micronuclei and cytosolic DNA leading to cGAS-STING activation. However, CIN and particularly aneuploidy can also facilitate immune evasion (36, 42). Analysis of CIN-type EG adenocarcinomas from TCGA revealed that immune-cold tumors with decreased CD8^+^ T-cell infiltration are enriched for *CCNE1* amplifications, suggesting that *CCNE1* amplification may also promote immune resistance in gastric cancer (19). It is important to note that TCGA samples predominantly represent primary tumors, whereas samples in our dataset primarily represent metastatic tumors which may have a distinct tumor-immune microenvironment. Interestingly, we did not observe any differences in T-cell abundance in *CCNE1*-amplified gastric cancer from transcriptomic analysis. However, there were changes in other immune cell populations including decreased B cells and neutrophils in *CCNE1*-amplified tumors, as well as increased M1 macrophages in *CCNE1*-amplified gastric adenocarcinoma. We also observed decreased T-cell inflammation scores and IFN signatures in *CCNE1*-amplified tumors. While these analyses are limited by extraction from bulk RNA sequencing, they suggest that *CCNE1*-amplified EGC may have a unique tumor-immune microenvironment that should be explored. Future studies such as single-cell RNA sequencing, spatial transcriptomics, or quantitative immunofluorescence may further define the immune cell composition and localization in *CCNE1*-amplified gastric cancer, and how the distinct tumor microenvironment may impact response to therapy.

Despite the large size of our dataset and clinical relevance, we acknowledge a few limitations within this study. There exists an extreme genetic and molecular heterogeneity of this patient cohort, for which TCGA subtypes of EG adenocarcinoma are well established (RRID:SCR_003193). While *CCNE1*-amplified tumors are enriched for alterations associated with CIN (such as *TP53* and *ERBB2*), molecular data in the CODEai database are aggregated, and so we are unable to assign TCGA subtypes and assess the relationship of *CCNE1* amplification on an individual sample basis. There was a limited number of *CCNE1*-amplified tumors having been treated with immunotherapy, as *CCNE1*-amplified tumors account for only 4%–7% of samples and not all samples have linked insurance claims data for real-world survival analysis. Also, some historical samples may have been obtained when immunotherapy was not approved, and patients with coamplification of *CCNE1* and *ERBB2* may have received trastuzumab alone prior to recent approval of chemotherapy in combination with trastuzumab and pembrolizumab per KEYNOTE-811 (4). As more patients with metastatic gastric cancer are receiving immunotherapy in the first-line setting in combination with chemotherapy (and trastuzumab for HER2^+^ tumors), it will be important to understand whether *CCNE1* amplification impacts the response to therapy in larger cohorts, as well as to identify other coalterations that may modulate the effect of *CCNE1* expression. We will need prospective data to assess whether treatment paradigms should be modified in the setting of *CCNE1* and *ERBB2* coamplification.

Overall, this study demonstrates that *CCNE1* amplification is associated with a distinct molecular profile in gastric cancer and may impact response to therapy, including targeted therapy and/or immunotherapy. While CCNE1 cannot yet be directly inhibited, several agents are under investigation to target *CCNE1* amplification through synthetic lethal strategies (43). Wee1 inhibitors have thus far been clinically limited in part due to toxicity (40), but ongoing trials are investigating *PKMYT1* inhibition (such as RP-6306; ref. 44) and *CDK2* inhibition (45) selectively in *CCNE1*-amplified cancers (46, 47). Thus, further investigation of *CCNE1* amplification as a predictive biomarker is warranted.

## Supplementary Material

Supplementary Figure S1 Supplementary Figure S1 shows the frequency of CCNE1 amplification in previously untreated and treated samples

Supplementary Figure S2 Supplementary Figure S2 shows frequently occurring molecular co-alterations in CCNE1-amplified EGC by histological subtype

Supplementary Figure S3 Supplementary Figure S3 shows frequently occurring molecular co-alterations in EA and GA with CCNE1 CN amplification or gain

Supplementary Figure S4 Supplementary Figure S4 shows co-altered genes with CCNE1 amplification from an EGC MSK-IMPACT sequencing cohort

Supplementary Figure S5 Supplementary Figure S5 shows immune cell infiltration in EGC with CCNE1 amplification or gain

Supplementary Figure S6 Supplementary Figure S6 shows differential gene expression analysis of CCNE1-amplified vs non-amplified EGC

Supplementary Figure S7 Supplementary Figure S7 shows treatment outcomes and survival analysis of CCNE1-amplified vs. non-amplified esophageal adenocarcinoma and esophagogastric junction carcinoma

Supplementary Table S1 Supplementary Table S1. Frequency of CCNE1 amplifications in EGC by primary tumor or metastatic site.
