# Supplementary material for: Molecular Landscape and Clinical Implication of CCNE1-amplified Esophagogastric Cancer
Source: Cancer Res Commun. 2024 Jun 3;4(6):1399–409. doi: 10.1158/2767-9764.CRC-23-0496 (PMC11146286; doi:10.1158/2767-9764.CRC-23-0496)
Supplement: Supplementary Figure S1 — shows the frequency of CCNE1 amplification in previously untreated and treated samples [file crc-23-0496-s01.pdf]

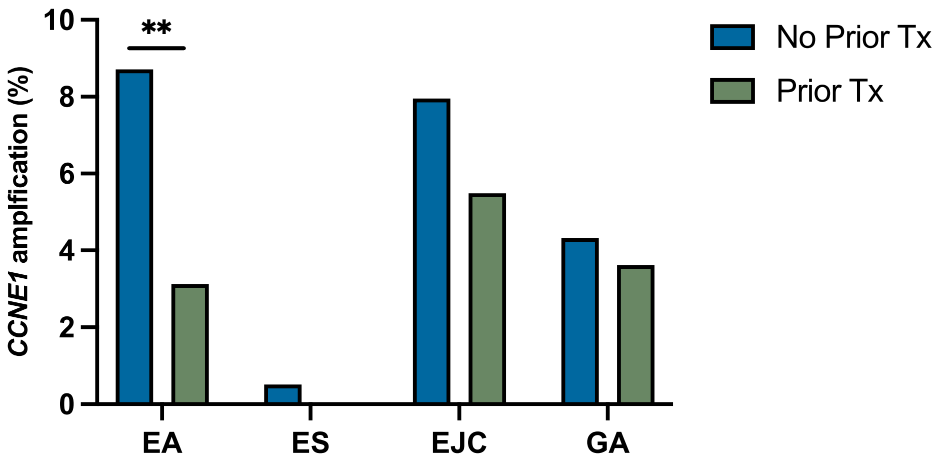

Supplementary Figure S1. Frequency of *CCNE1* amplification in previously untreated and treated samples.

Frequency of *CCNE1* amplification in EGC samples obtained from tumor samples with no prior systemic treatment (Tx) or prior treatment. Statistical significance is displayed as the following: **p < 0.01
