# Supplementary material for: Molecular Landscape and Clinical Implication of CCNE1-amplified Esophagogastric Cancer
Source: Cancer Res Commun. 2024 Jun 3;4(6):1399–409. doi: 10.1158/2767-9764.CRC-23-0496 (PMC11146286; doi:10.1158/2767-9764.CRC-23-0496)
Supplement: Supplementary Figure S2 — shows frequently occurring molecular co-alterations in CCNE1-amplified EGC by histological subtype [file crc-23-0496-s02.pdf]

A

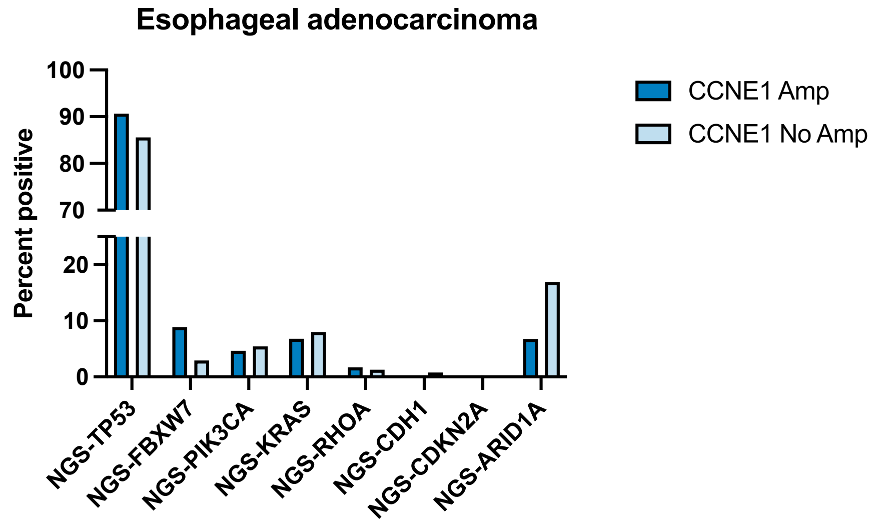

B

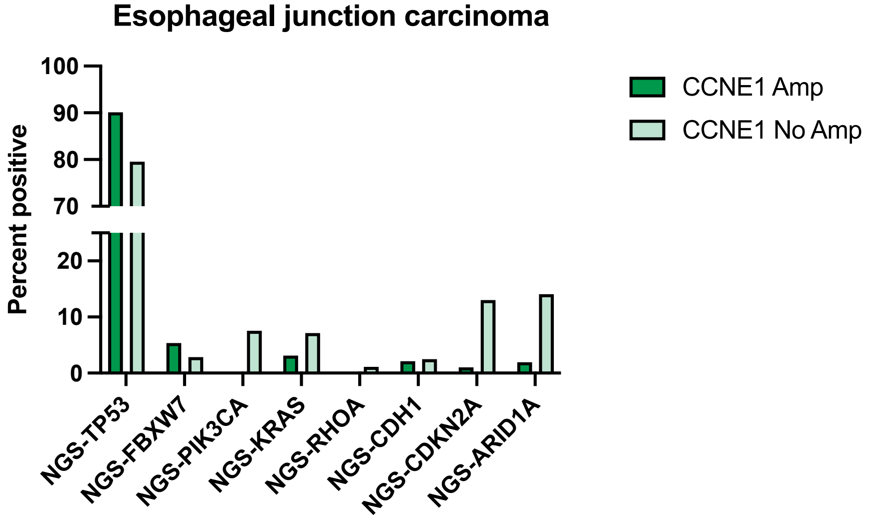

C

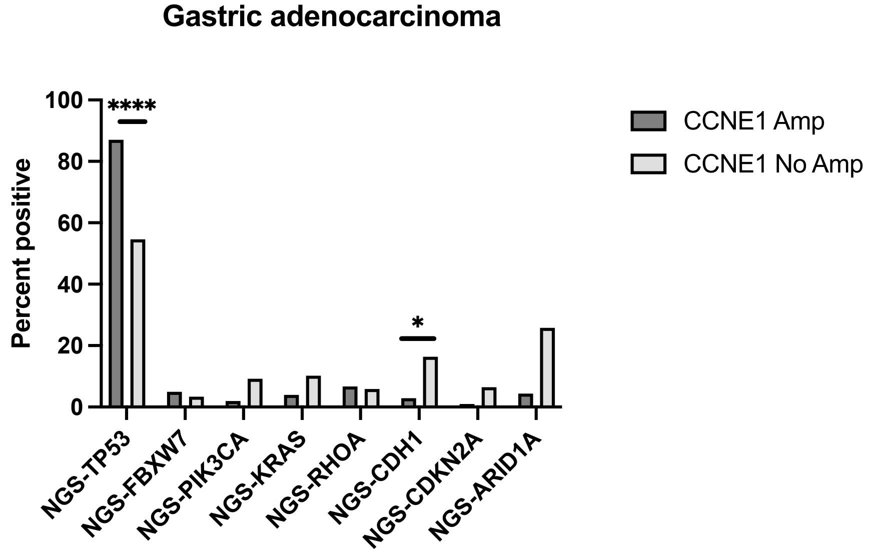

Supplementary Figure S2. Frequently occurring molecular co-alterations in *CCNE1*-amplified EGC by histological subtype

Frequency of co-mutated genes among *CCNE1*-amplified and non-amplified EA by WES and NGS (A). Frequency of co-mutated genes among *CCNE1*-amplified and non-amplified EJC by WES and NGS (B). Frequency of co-mutated genes among *CCNE1*-amplified and non-amplified GA by WES and NGS (C). Statistical significance is displayed as the following: * q<0.05; **** q<0.0001.
