# Supplementary material for: Molecular Landscape and Clinical Implication of CCNE1-amplified Esophagogastric Cancer
Source: Cancer Res Commun. 2024 Jun 3;4(6):1399–409. doi: 10.1158/2767-9764.CRC-23-0496 (PMC11146286; doi:10.1158/2767-9764.CRC-23-0496)
Supplement: Supplementary Figure S3 — shows frequently occurring molecular co-alterations in EA and GA with CCNE1 CN amplification or gain [file crc-23-0496-s03.pdf]

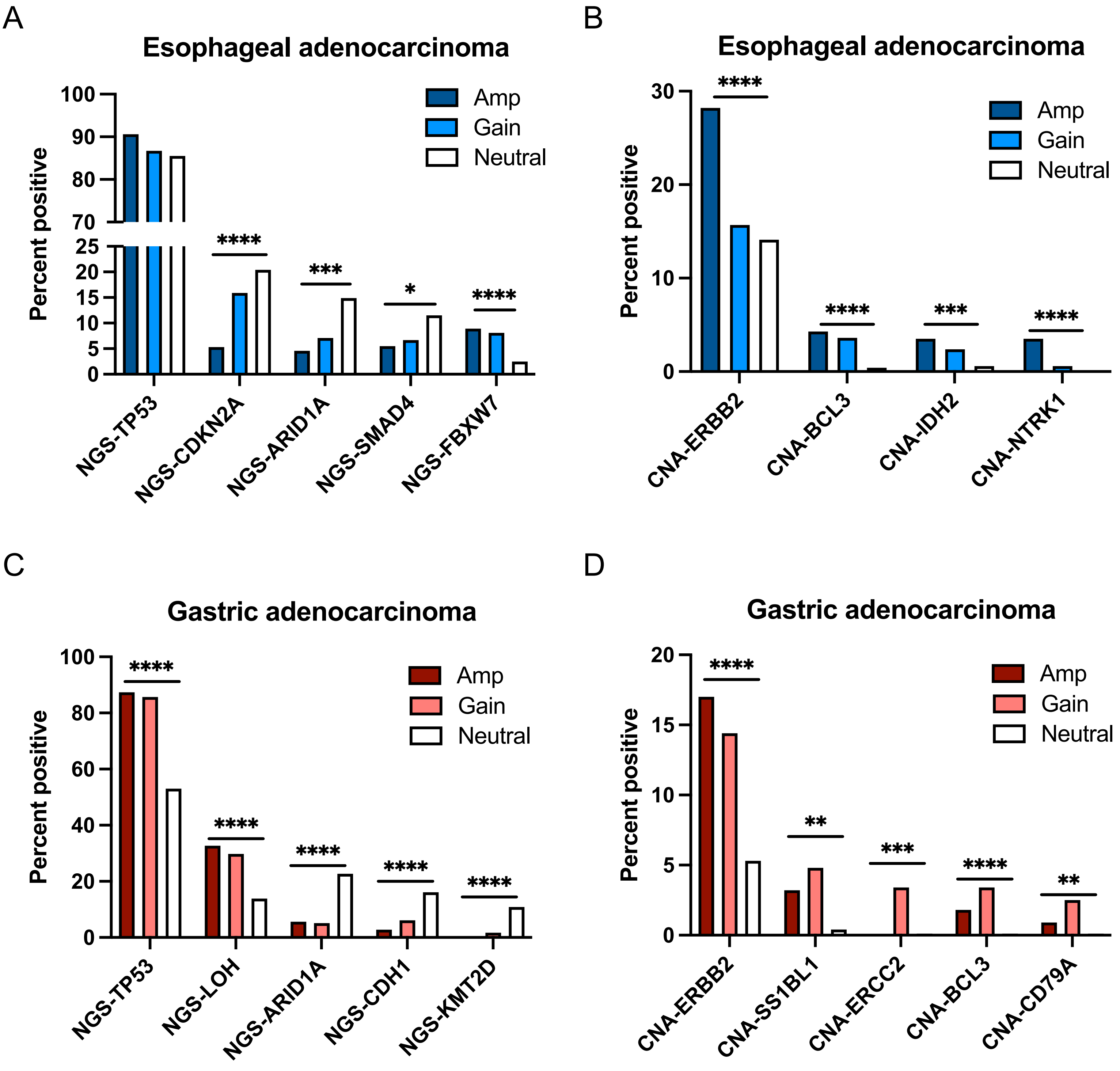

Supplementary Figure S3. Frequently occurring molecular co-alterations in EA and GA with *CCNE1* CN amplification or gain.

Frequency of co-mutated genes among EA samples with *CCNE1* amplification (CN ≥6), gain (CN ≥3 and <6), and neutral CN (CN <3) (A). Frequency of copy number alterations among *CCNE1* CN cohorts in EA (B). Frequency of co-mutated genes among *CCNE1* CN cohorts in GA (C).). Frequency of copy number alterations among *CCNE1* CN cohorts in GA (D). Statistical significance is displayed as the following: * q<0.05; ** q<0.01; *** q<0.001; **** q<0.0001.
