# Supplementary material for: Molecular Landscape and Clinical Implication of CCNE1-amplified Esophagogastric Cancer
Source: Cancer Res Commun. 2024 Jun 3;4(6):1399–409. doi: 10.1158/2767-9764.CRC-23-0496 (PMC11146286; doi:10.1158/2767-9764.CRC-23-0496)
Supplement: Supplementary Figure S4 — shows co-altered genes with CCNE1 amplification from an EGC MSK-IMPACT sequencing cohort [file crc-23-0496-s04.pdf]

A

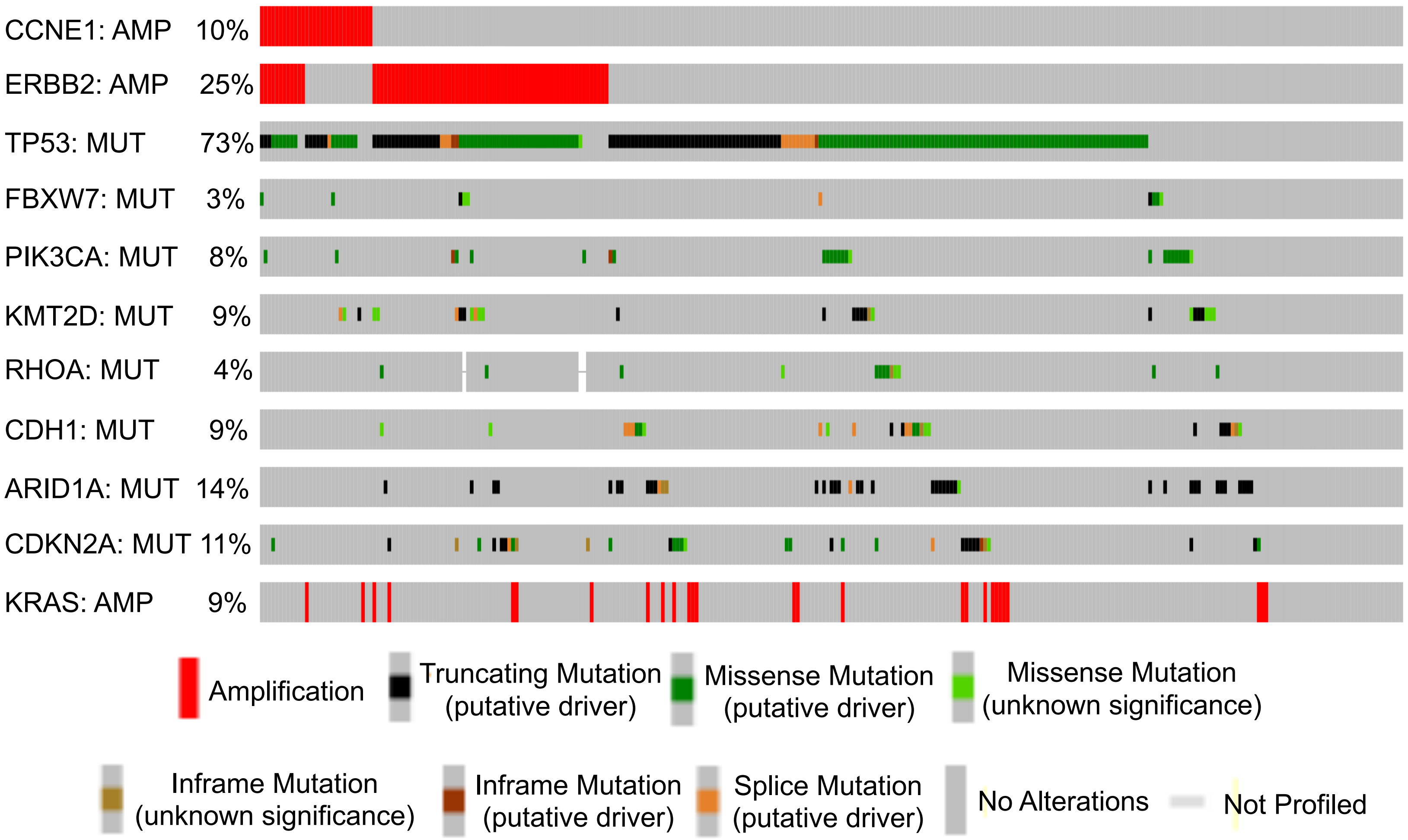

B

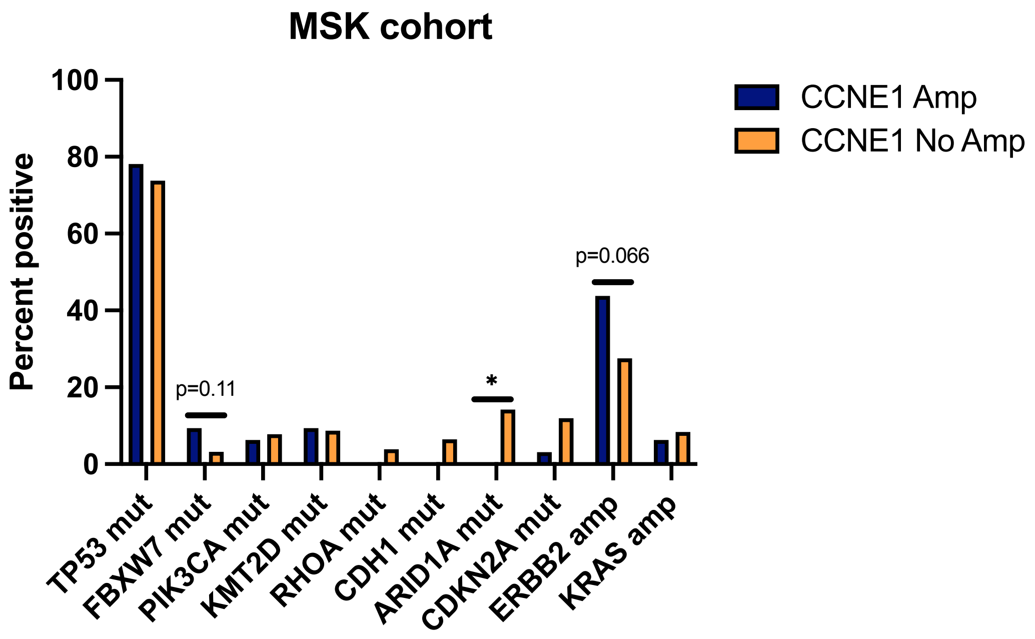

Supplementary Figure S4. Co-altered genes with CCNE1 amplification from an EGC MSK-IMPACT sequencing cohort.

Oncoprint of select genes comparing CCNE1-amplified vs. non-amplified EG adenocarcinoma from Janjigian et al, Cancer Discovery 2018 (A). Frequency of select co-altered genes in CCNE1-amplified vs. non-amplified EG adenocarcinoma (B).
