# Supplementary material for: Molecular Landscape and Clinical Implication of CCNE1-amplified Esophagogastric Cancer
Source: Cancer Res Commun. 2024 Jun 3;4(6):1399–409. doi: 10.1158/2767-9764.CRC-23-0496 (PMC11146286; doi:10.1158/2767-9764.CRC-23-0496)
Supplement: Supplementary Figure S5 — shows immune cell infiltration in EGC with CCNE1 amplification or gain [file crc-23-0496-s05.pdf]

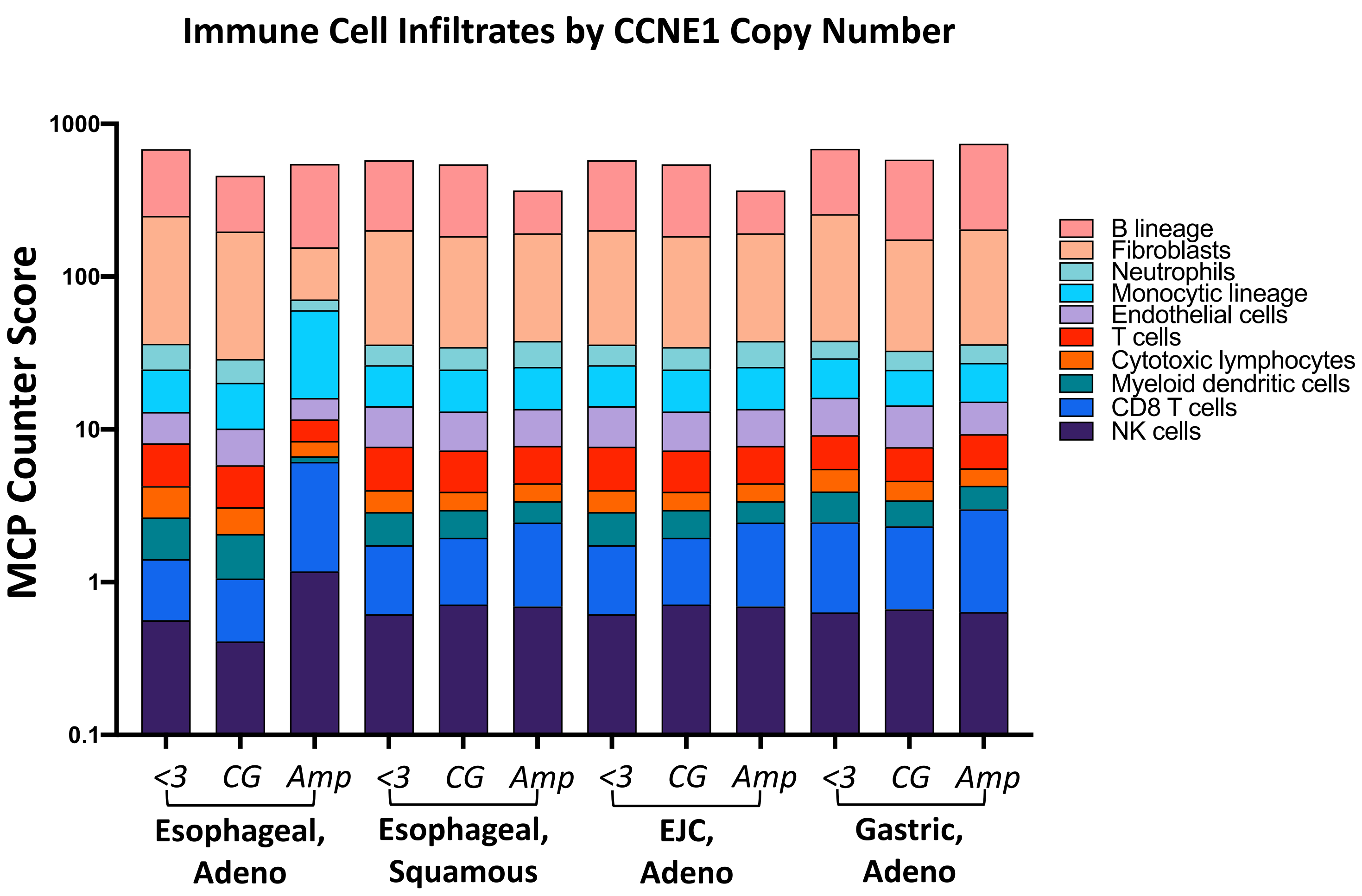

Supplementary Figure S5: Immune cell infiltration in EGC with CCNE1 amplification or gain. Immune cell infiltration extrapolated from WTS and RNA deconvolution analysis in EGC with CCNE1 amplification (Amp) , CCNE1 gain (CG), or neutral CN (<3).
