# Supplementary material for: Molecular Landscape and Clinical Implication of CCNE1-amplified Esophagogastric Cancer
Source: Cancer Res Commun. 2024 Jun 3;4(6):1399–409. doi: 10.1158/2767-9764.CRC-23-0496 (PMC11146286; doi:10.1158/2767-9764.CRC-23-0496)
Supplement: Supplementary Figure S6 — shows differential gene expression analysis of CCNE1-amplified vs non-amplified EGC [file crc-23-0496-s06.pdf]

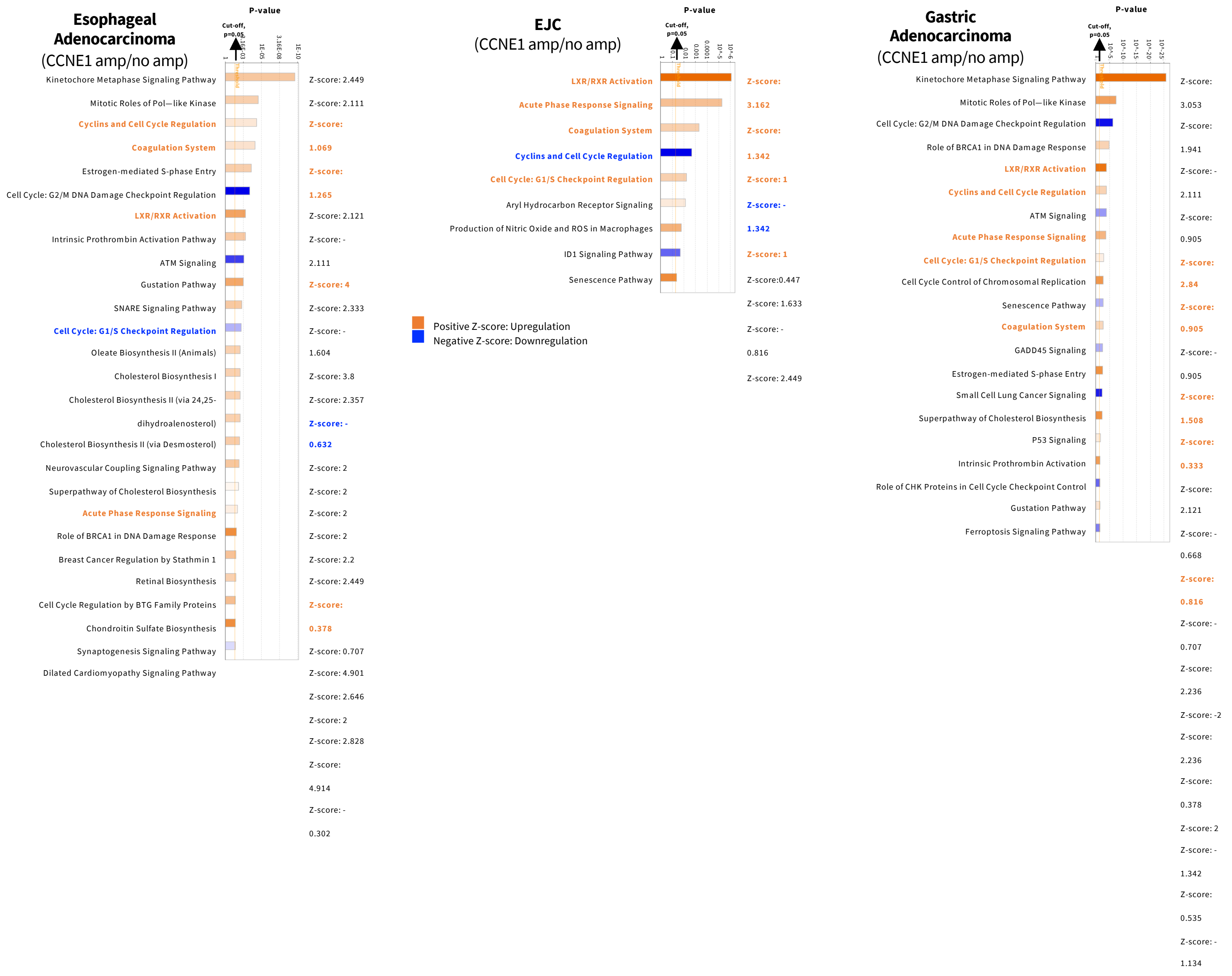

Supplementary Figure S6: Differential gene expression analysis of *CCNE1*-amplified vs non-amplified EGC. Ingenuity pathway analysis (IPA) from WTS of *CCNE1*-amplified vs. non-amplified EA, EGC, and GA.
