# Supplementary material for: Molecular Landscape and Clinical Implication of CCNE1-amplified Esophagogastric Cancer
Source: Cancer Res Commun. 2024 Jun 3;4(6):1399–409. doi: 10.1158/2767-9764.CRC-23-0496 (PMC11146286; doi:10.1158/2767-9764.CRC-23-0496)
Supplement: Supplementary Figure S7 — shows treatment outcomes and survival analysis of CCNE1-amplified vs. non-amplified esophageal adenocarcinoma and esophagogastric junction carcinoma [file crc-23-0496-s07.pdf]

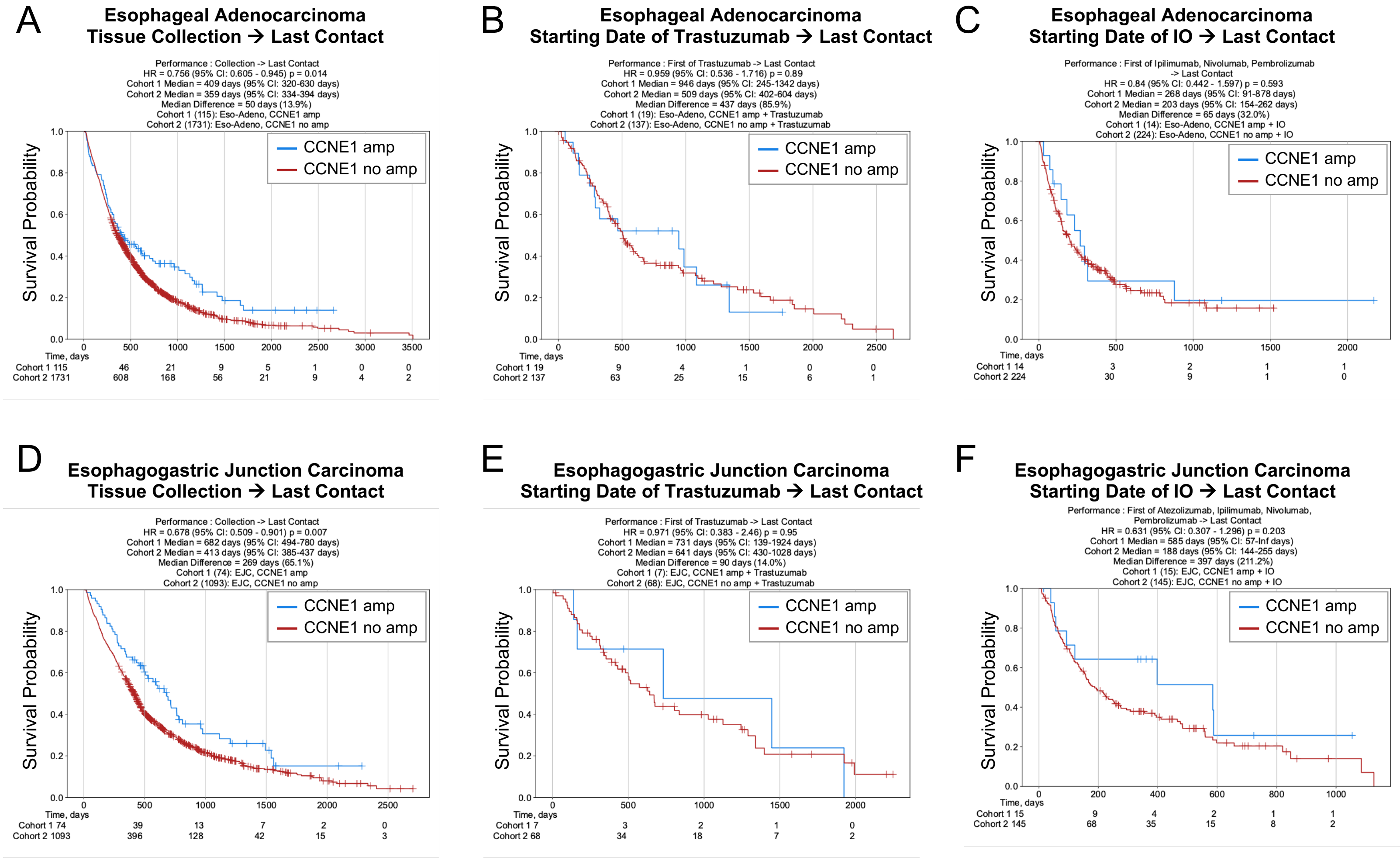

Supplementary Figure S7. Treatment outcomes and survival analysis of *CCNE1*-amplified vs. non-amplified esophageal adenocarcinoma and esophagogastric junction carcinoma.
Overall survival (calculated from tissue collection to last day of contact) of *CCNE1*-amplified vs. non-amplified EA (A). Comparison of survival of *CCNE1*-amplified vs non-amplified EA treated with trastuzumab (calculated from start of treatment to last day of contact) (B). Comparison of survival of *CCNE1*-amplified vs non-amplified EA treated with immunotherapy with a PD1 or PD-L1 inhibitor (C). Overall survival (calculated from tissue collection to last day of contact) of *CCNE1*-amplified vs. non-amplified EJC (D). Comparison of survival of *CCNE1*-amplified vs non-amplified EJC treated with trastuzumab (E). Comparison of survival of *CCNE1*-amplified vs non-amplified EJC treated with immunotherapy with a PD1 or PD-L1 inhibitor (F).
