# Supplementary material for: Molecular Landscape and Clinical Implication of CCNE1-amplified Esophagogastric Cancer
Source: Cancer Res Commun. 2024 Jun 3;4(6):1399–409. doi: 10.1158/2767-9764.CRC-23-0496 (PMC11146286; doi:10.1158/2767-9764.CRC-23-0496)
Supplement: Supplementary Table S1 — Frequency of CCNE1 amplifications in EGC by primary tumor or metastatic site. [file crc-23-0496-s08.docx]

**Supplementary Table S1. Frequency of CCNE1 amplifications in EGC by primary tumor or metastatic site.**

| **Site** | **EA** | | **ES** | | **EJC** | | **GA** | |
| --- | --- | --- | --- | --- | --- | --- | --- | --- |
|  | **Pos/Total** | **Amp %** | **Pos/Total** | **Amp %** | **Pos/Total** | **Amp %** | **Pos/Total** | **Amp %** |
| C/ST | 5/31 | 16.10% | 0/8 | 0% | 2/23 | 8.70% | 19/153 | 3.33% |
| Mixed | 3/23 | 13.00% | 0/4 | 0% | 1/16 | 6.25% | 2/29 | 7% |
| Brain | 5/50 | 10% | 0/1 | 0% | 2/13 | 15.40% | 0/6 | 0.00% |
| Lymph Node | 10/105 | 9.52% | 1/47 | 2.13% | 8/71 | 11.30% | 8/77 | 10% |
| Liver | 17/193 | 8.81% | 0/33 | 0% | 13/137 | 9.49% | 19/172 | 11.05% |
| Non-Liver GI | 13/165 | 7.88% | 0/21 | 0% | 34/446 | 7.62% | 6/215 | 2.80% |
| Lung | 6/79 | 7.59% | 0/39 | 0% | 0/28 | 0% | 1/27 | 4% |
| Primary GI | 79/1497 | 5.28% | 4/561 | 0.71% | 40/650 | 6.15% | 68/1732 | 4% |
| Bone | 2/38 | 5.26% | 0/10 | 0% | 0/13 | 0% | 1/58 | 5.26% |
| Other | 2/40 | 5.00% | 1/23 | 4.35% | 0/18 | 0% | 1/28 | 3.60% |
| Breast | 0/3 | 0% | 0/0 | - | - | - | 0/1 | 0.00% |
| C/CW | 0/3 | 0% | 0/0 | - | - | - | - |  |
| GU | 0/3 | 0% | 0/0 | - | 0/1 | 0% | 0/7 | 0.00% |
| GYN | 0/3 | 0% | 0/0 | - | 1/5 | 20% | 0/52 | 0.00% |
| O/P/R | 0/34 | 0% | 0/4 | 0% | 0/26 | 0% | 1/175 | 0.57% |
| Skin | 0/9 | 0% |  | - | 0/2 | 0% | 0/7 | 0% |
